# Effects of Dapagliflozin on the Bone of Patients with CKD on Dialysis

**DOI:** 10.1007/s00223-026-01587-7

**Published:** 2026-08-14

**Authors:** Lauter E. Pelepenko, Joaquim Barreto, Marília P. Martins, Pedro B. Chiteculo, Jessica M. Camargo, Mariana C. de Oliveira, Eric A. Corrêa, Mauro Pascoa, Gil Guerra-Junior, Wagner Vasques Dominguez, Cinthia E. M. Carbonara, Loïc Louvet, Rosa M. A. Moyses, Makoto Kuro-O, Andrei C. Sposito, Rodrigo Bueno de Oliveira

**Affiliations:** 1 Laboratory for Evaluation of Mineral and Bone Disorder in Nephrology (LEMON), Nephrology Division, School of Medical Sciences, University of Campinas (Unicamp), Rua Cinco de Junho, 350, Cidade Universitária Zeferino Vaz – UNICAMP, Campinas, SP 13083-877 Brazil; 2 Laboratory of Atherosclerosis and Vascular Biology, Cardiology Division, School of Medical Sciences, University of Campinas (Unicamp), Campinas, Brazil; 3 Growth and Development Laboratory (LabCreD), Center for Investigation in Pediatrics, School of Medical Sciences, University of Campinas (Unicamp), Campinas, Brazil; 4 Laboratório de Fisiopatologia Renal (LIM 16), Nephrology Department, Hospital das Clínicas da Faculdade de Medicina da Universidade de São Paulo (HCFMUSP), University of São Paulo (USP), São Paulo, Brazil; 5 Charles and Jane Pak Center for Mineral Metabolism and Clinical Research, University of Texas Southwestern Medical Center, Dallas, TX USA; 6 MP3CV “Pathophysiological Mechanisms and Consequences of Cardiovascular Calcifications” Laboratory, UPJV UR7517, University of Picardie Jules Verne, Avenue Laennec, Amiens, France; 7 Division of Antiaging Medicine, Center for Molecular Medicine, Jichi Medical University, Shimotsuke, Tochigi Japan

**Keywords:** Bone, Chronic kidney disease, Dapagliflozin, Dialysis, Klotho, SGLT2

## Abstract

The effects of gliflozins on bones of CKD patients on dialysis are unknown. Recently, SGLT2 expression in bone tissue of patients with CKD has been reported. We hypothesized that dapagliflozin may act in bone cells and modulate the Klotho-FGF23 axis. This study analyzed the effects of dapagliflozin on serum bone biomarkers and related outcomes. In this predefined post-hoc analysis of a previous RCT, patients on dialysis received (1:1) dapagliflozin 10 mg daily or standard care for 24 weeks. The primary endpoint was the change from baseline in serum Klotho and FGF23 levels, compared by ranked analysis of covariance, adjusted by baseline. Exploratory analyses evaluated calcium, phosphate, PTH, bone-ALP, TRAP-5b, DKK1, sclerostin, and bone proteins. Bone fractures, osteopenia, and osteoporosis were outcomes of interest. Seventy-nine patients were included in this analysis (Control N=40 and Dapagliflozin N=39). At baseline, no significant differences in biomarkers were observed. The prevalence of osteopenia and osteoporosis was 73% and 43% (*p* = 0.60 and *p* = 1.00), respectively. After 24 weeks, the between-group analysis of the change from baseline (delta), adjusted by baseline, revealed a significant difference in serum Klotho [19.1 (− 38.9–86.3) pg/mL, Control group; − 29.1 (− 94.1–43.9) pg/mL, Dapagliflozin group (F:4.946, *p* = 0.03)], but not on FGF23 levels, nor other biomarkers. The follow-up prevalences of osteopenia and osteoporosis were 70% and 48% (*p* = 0.27 and *p* = 0.61, respectively). No bone fractures were observed. Dapagliflozin use over 24 weeks in patients on dialysis was independently associated with lowered serum Klotho levels, without effects in other bone biomarkers or related outcomes.

**Supplementary Information:**

The online version contains supplementary material available at 10.1007/s00223-026-01587-7.

## Introduction

Sodium-glucose cotransporter-2 inhibitors (SGLT2i, or gliflozins) are currently considered a cornerstone of treatment for chronic kidney disease (CKD) and CKD-associated cardiovascular disease (CVD) [1]. The cardiorenal protective effects of these medications are consistently supported by numerous large-scale outcome trials [2–12]. The mineral and bone disorder associated with CKD (CKD-MBD) add several non-traditional cardiovascular risk factors. For example, severe hyperphosphatemia directly promotes vascular calcification and endothelial dysfunction. Furthermore, the reduction of vitamin K levels in this population exacerbates vascular stiffness by impairing the activation of matrix Gla proteins, a critical calcification inhibitor [13, 14]. These combined disturbances increase cardiovascular morbidity in dialysis patients.

The effects of gliflozin on bone metabolism and related outcomes in patients with CKD are not well understood. Although initial concerns about an increased risk of bone adverse effects associated with gliflozins [15–18] have been dismissed by subsequent studies [2, 5, 6, 8, 19], there is still limited information on patients with moderate to advanced CKD. A systematic review [20] of the effects of gliflozins on bone in patients with CKD indicated a possible reassuring bone safety profile, even though the included studies mainly involved patients with early-stage CKD and diabetes. Uncertainty remains in the population with CKD on dialysis.

Dialysis patients face a high risk of fractures, with distinct pathophysiological and clinical profiles characterizing hip *versus* vertebral events. While hip fractures often present acute, catastrophic structural failures resulting from falls, vertebral fractures frequently occur silently, accumulating progressively due to impaired trabecular microarchitecture. Managing vertebral fractures in advanced CKD requires specialized paradigms, as outlined in the recent consensus statement [21]. Distinguishing these fracture types remains vital when evaluating how novel therapies like gliflozins influence skeletal integrity.

The concern about the effects of gliflozins on the skeleton can be justified for several reasons. Patients on dialysis constitute a CKD population at the highest risk for bone disorders and fractures [22]. Bone tissue demands approximately 10 to 15% of the cardiac output, and presents a significant vascular endothelium net, in which bone cells highly depend on glucose [23]. Common pathophysiological pathways between bone and the cardiovascular system, such as the Klotho-FGF23 axis, apoptosis, and fibrosis pathways in the heart [24], are highly affected in advanced CKD and might be influenced by gliflozins through their non-renal-mediated mechanisms [25, 26].

Evidence from experimental and clinical studies indicates a link between gliflozins and the modulation of Klotho, providing insights into the protective mechanisms of gliflozins in various diseases, such as diabetes and cardiorenal disease [27–29]. Although the Klotho-FGF23 axis is involved in bone mineralization, there is no information on the effects of gliflozins on this process.

Recently, our group reported that bone tissue from healthy individuals and patients with CKD consistently expresses the gene SLC5A2 and its encoded protein SGLT2 [30]. It remains unknown whether long-term interaction of gliflozins with the SGLT2 protein expressed by bone cells would have any modulatory effect on the Klotho-FGF23 axis, or whether it would affect any bone-related outcomes in patients with CKD on dialysis.

Currently, there is data on the pharmacokinetic of dapagliflozin in patients with CKD on dialysis showing that dapagliflozin is safe, slightly dialyzable, and does not accumulate [31]. In a recent randomized controlled trial (RCT) [32], the safety of dapagliflozin and the hypothesis that it improves cardiac function, exercise capacity, and heart failure symptom burden in patients on dialysis were tested. No metabolic issues nor concerns regarding cardiovascular safety outcomes were identified during 24 weeks of dapagliflozin treatment.

This body of evidence makes it plausible to analyze the effects of gliflozins on bone tissue in patients with CKD on dialysis. However, because dapagliflozin’s primary action requires glomerular filtration, its classic glycosuric effects may be neutralized in patients lacking residual renal function. This altered physiological environment could raise concerns about the potential systemic complications and unexpected actions on non-renal tissues, including the skeleton [33]. In this sense, the distinct challenges of utilizing antidiabetic therapies in advanced CKD require a cautious evaluation of their bone-health impacts in anuric populations.

This study aimed to perform a *post-hoc* analysis of the quoted RCT data [32] to assess the effects of 24-week treatment with dapagliflozin in patients on dialysis on serum Klotho and FGF23 levels. As secondary analyses, a broad panel of serum bone biomarkers and related clinical outcomes, including fractures, prevalence of osteopenia and osteoporosis, were also analyzed. In this study, we hypothesized that dapagliflozin may affect the serum Klotho and FGF23 levels either directly through SGLT2 interaction in osteocytes or indirectly via other mineral metabolic pathways.

## Methods

This is a pre-specified post hoc analysis of an RCT that has been previously reported [32]. Briefly, 80 clinically stable patients with CKD on dialysis were evaluated in this prospective, randomized, and open-label study, performed between January 2023 and August 2024. The trial strictly followed the Good Clinical Practice guidelines, applicable regulatory requirements, and Declaration of Helsinki principles. The institutional review board approved each enrolled site before the study initiation, and all participants signed an informed consent form. The study protocol was previously published [34] and registered at Clinicaltrials.gov (NCT05685394) before recruitment.

Participants ≥ 18 years old, and on regular dialysis for more than three months were enrolled and randomized. Exclusion criteria were liver failure, pregnancy, allergies to the study drug, and recent cardiovascular events (defined as decompensated heart failure or acute coronary syndrome within three months before screening). All participants were required to demonstrate a clear capacity for informed consent and to maintain a stable dialysis regimen with no planned modifications within six months of enrollment.

Participants were randomized 1:1 to receive dapagliflozin 10 mg orally once daily alongside standard treatment (Dapagliflozin group) or standard treatment alone (Control group) for 24 weeks. The standard treatment was defined as the prescription determined by their attending nephrologist (including those related to the regular dialytic treatment) without any interference from study personnel. The Research Electronic Data Capture [REDCap] system was used for data registration.

### Laboratory Measurements

Peripheral blood samples were collected at baseline and at week 24 under standardized conditions, processed by centrifugation at 3500 rpm for 10 min at 4°C to separate serum, followed by aliquoting and storage at −80°C until analysis.

Investigators were blinded to group allocation until completion of all the serum analyses, which included measurements of serum total calcium (TCa), phosphate (P), parathyroid hormone (PTH), Klotho, fibroblast growth factor-23 (FGF23), bone alkaline phosphatase (bALP), tartrate-resistant acid phosphatase type−5b (TRAP5b), Dickkopf-1 (DKK1), sclerostin, osteopontin, osteoprotegerin, and osteocalcin levels.

MILLIPLEX® MAP Human Bone Magnetic Bead Panels (Cat. #HBNMAG-51K; Millipore, Burlington, MA, USA) were employed for analyses of serum FGF23, DKK1, sclerostin, osteopontin, osteoprotegerin, and osteocalcin levels strictly according to the manufacturer’s instructions. Briefly, 25 µL of standards, controls, and samples were incubated overnight at 4°C with antibody-immobilized magnetic beads. Following washes, plates were incubated with the detection antibodies for one hour at room temperature, followed by a 30-min incubation with streptavidin–phycoerythrin. Data was acquired on a Luminex® MAGPIX® and analyzed using MILLIPLEX® Analyst software with a 5-parameter logistic curve fit.

Additionally, assays (Immutopics, Quidel, USA) for PTH (code 60-3000 human PTH 1-84 EIA; reference range from 16 to 256 pg/mL), bone alkaline phosphatase (code 8012 BAP EIA; reference range from 11.9 to 88 U/L, intra assay 3.9–5.8% and inter assay 5.0–7.6%), TRAP5b (code 8033 TRAP5b EIA; reference range from 3.1 to 9.8 U/L, intra assay 1.9–2.2% and inter assay 2.0–3.0%) were evaluated by enzymatic immunoassays according to manufacturer instructions. Also, the assay (Immuno-Biological Laboratories Co, IBL, USA) for soluble Klotho (code JP27998; measurement range from 93.75 to 6000 pg/mL) was also evaluated by enzymatic immunoassays according to manufacturer instructions. Intra- and inter-assay percentages for this assay were 3.1–3.5% and 2.9–11.4%, respectively.

### Study Endpoints

The predefined endpoint was the influence of dapagliflozin administered for 24 weeks on serum Klotho and FGF23 levels. Exploratory analyses were further performed involving a panel of bone biomarkers and bone-related clinical outcomes, including serum TCa, P, PTH, bALP, TRAP5b, DKK1, sclerostin, osteopontin, osteoprotegerin, and osteocalcin levels measured at baseline and follow-up, alongside the outcomes bone fractures, osteopenia, and osteoporosis prevalence. Dual-energy X-ray absorptiometry (DEXA) parameters were obtained at baseline and after 24 weeks for bone mineral density (BMD, in g/cm^2^) and the corresponding T-score at multiple anatomical sites. The lowest T-score obtained from these locations was considered for osteopenia and osteoporosis diagnosis.

### Statistical Analysis

Data were presented as mean and standard deviation (SD) or median and 25th and 75th percentiles for continuous variables, or as numbers and percentages for categorical data. Data distribution normality was assessed using the Kolmogorov–Smirnov test. Outliers were detected using the interquartile range (IQR) method. Extreme outliers [i.e., values ≥ (3 × IQR) + Q3] were excluded from the analyses and replaced by the values corresponding to the 95th percentile (winsorization of 5%). A sensitivity analysis was performed to confirm the observed results and to avoid bias. The corresponding number of samples analyzed for the predefined endpoint is indicated in the results.

Comparisons of means between continuous variables, skewed data, and categorical variables were performed using the Student’s t-test, Mann–Whitney, McNemar, or chi-squared tests. The mean (or median) values of each parameter at baseline were compared within each group using the paired t-test or the Wilcoxon signed-rank test.

The delta variation of serum Klotho and FGF23 levels between the end of follow-up and baseline was compared using ranked analysis of covariance (RANCOVA) to assess the potential effect of dapagliflozin, influenced by other factors or covariates, and adjusted by its respective baseline value. The delta variation of Klotho or FGF23 was used as the dependent variable, the study group (Control or Dapagliflozin) as a fixed factor, and its corresponding baseline value as a covariate for adjustment. The adjusted median difference between the variations was calculated by the Hodges-Lehman analysis. Complementary adjustments in the RANCOVA models considered relevant factors or covariates related to Klotho and FGF23 modulation, such as “age”, “anuria”, “sex”, and ranked “baseline phosphate”, “baseline FGF23”, or “baseline Klotho”. No further adjustments for multiple comparisons were applied to the exploratory endpoints.

All statistical analyses were performed using IBM SPSS Statistics version 30.0 using a two-sided *p*-value <0.05 significance.

## Results

### Baseline Studies

One participant randomized to the dapagliflozin group did not complete baseline assessments; therefore, data from 79 patients were analyzed.

Overall, the sample was predominantly constituted by white males, with 18% diabetes *mellitus* prevalence. Anuria was observed in 41 (52%) patients, with no significant differences between the groups (*p* = 0.50). The diagnosis of osteopenia and osteoporosis at baseline occurred in 55 (73%) and 32 (43%) patients, being similar between groups (Control, 30 vs. Dapagliflozin, 25, *p* = 0.60; and Control, 17 vs. Dapagliflozin, 15, *p* = 1.00, respectively). Biochemical profiles related to mineral and bone metabolism were comparable between the Control and the Dapagliflozin groups, without statistically significant differences. The baseline demographic, clinical, and bone-related biomarkers characteristics of the participants are detailed in Table 1.

**Table 1:** Demographic, clinical, and biochemical parameters at baseline for the control and the dapagliflozin groups

	All	Control	Dapagliflozin	*p*
Parameter	(N = 79)	(N = 40)	(N = 39)	
Age (years)	56 ± 16	58 ± 14	54 ± 17	0.28
Body mass index (kg/m^2^)	25 (23–28)	26 (23–28)	24 (22–28)	0.24
Gender (male; N, %)	43 (54)	21 (52)	22 (56)	0.73
White (N, %)	48 (61)	27 (67)	21 (54)	0.21
Diabetes *mellitus* (N, %)	14 (18)	5 (12)	9 (23)	0.25
*CKD etiology (N, %)*				0.58
Arterial hypertension	30 (38)	14 (35)	16 (41)	
Chronic GN	9 (11)	5 (12)	4 (10)	
Diabetes *mellitus*	12 (15)	4 (10)	8 (20)	
Hemodialysis (N, %)	67 (85)	31 (77)	36 (92)	0.11
Dialysis vintage (years)	3.5 (1.7–6.5)	3.5 (1.4–8.4)	3.5 (2.3–6.1)	0.62
*CKD-MBD-related drugs*				
Calcium Carbonate (N, %)	16 (20)	7 (17)	9 (23)	0.73
Colecalciferol (N, %)	18 (23)	12 (30)	6 (15)	0.2
Calcitriol (N, %)	7 (9)	6 (15)	1 (3)	0.12
Sevelamer (N, %)	47 (59)	26 (65)	21 (54)	0.43
Cinacalcet (N, %)	25 (32)	13 (32)	12 (31)	1.0
*Bone-related biomarkers*				
Klotho (pg/mL)	561 (431–786)	587 (412–842)	552 (442–662)	0.36
FGF23 (pg/mL)	553 (186–2393)	486 (145–2610)	597 (203–2012)	0.87
Total calcium (mg/dL)	9.4 (9–9.8)	9.2 (8.9–9.7)	9.6 (9.1–9.8)	0.40
Phosphate (mg/dL)	4.3 (3.3–5.2)	4.2 (3.5–5.0)	4.3 (3.3–5.3)	0.59
PTH (pg/mL)	80 (32–185)	108 (31–191)	65 (41–159)	0.41
Bone_ALP (IU/L)	33 (21–60)	31 (22–58)	36 (19–65)	0.93
TRAP5b (IU/L)	8.6 (5.9–12.7)	8.2 (6–13)	8.7 (5.8–11.6)	0.95
DKK1 (pg/mL)	599 (452–965)	504 (454–925)	654 (452–1008)	0.30
Sclerostin (pg/mL)	1650 (1206–2259)	1514 (1097–2169)	1780 (1259–2517)	0.51
Osteopontin (pg/mL)	52,214 (34,597–73,841)	52,214 (40,482–64,842)	52,214 (32,421–80,524)	0.88
Osteoprotegerin (pg/mL)	842 (459–1270)	908 (537–1270)	824 (412–1243)	0.31
Osteocalcin (pg/mL)	81,788 (44,595–143,640)	83,755 (45,604–146,887)	81,788 (40,894–143,640)	0.96

CKD, chronic kidney disease; GN, glomerulonephritis; FGF23, fibroblast growth factor-23; PTH, parathyroid hormone; ALP, alkaline phosphatase; TRAP5b, tartrate-resistant acid phosphatase type−5b; DKK1, Dickkopf-1. Data were expressed as mean ± standard deviation, median, and 25th and 75th percentiles, or number and percentage. Comparisons of means or medians between groups were made using the independent samples t-test, the Mann–Whitney test, or the chi-square test

### Post-Treatment Studies

At follow-up, 12 patients were excluded [8 (67%) in the Control group and 4 (33%) in the Dapagliflozin group; *p* = 0.35], resulting in 67 (85%) patients who completed the study treatment and were included in the analysis. The reasons for exclusion were loss of follow-up in 7 (58%), kidney transplantation in 3 (25%), and death in 2 (17%) patients in the control group. Patients who were excluded from analysis had similar baseline characteristics.

#### Effects of Dapagliflozin Treatment on Serum Klotho and FGF23 Levels

The intragroup comparison of means of serum Klotho levels at baseline and after 24 weeks showed no significant differences: 587 (412–842) vs. 619 (429–796) pg/mL, *p* = 0.23 in the Control group; 552 (442–662) vs. 501 (404–673) pg/mL, *p* = 0.17 in the Dapagliflozin group.

The intragroup comparison of means of serum FGF23 levels at baseline and after 24 weeks showed no significant differences: 486 (145–2610) vs. 431 (186–1805) pg/mL, *p* = 0.55 in the Control group; 597 (203–2012) vs. 750 (198–2634) pg/mL, *p* = 0.53 in the Dapagliflozin group.

**Fig. 1:**
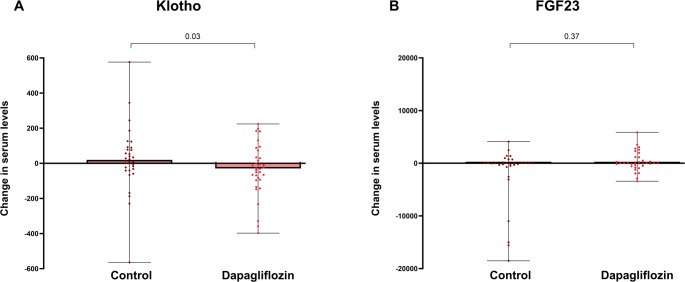
**A** Change in serum Klotho levels in dialysis patients treated or not with dapagliflozin for 24 weeks (N = 66); **B** Change in serum FGF23 levels in dialysis patients treated or not with dapagliflozin for 24 weeks (N = 65). Statistical comparison between treatment groups was performed by RANCOVA adjusted for their corresponding baseline values

The analysis between groups of the variation of Klotho and FGF23 between the end of the follow-up and its baseline (delta), adjusted for its respective baseline values, demonstrated the influence of dapagliflozin on serum Klotho levels but not on FGF23 (Fig. 1).

The variation in serum Klotho levels between groups was 19.1 (− 38.9 to 86.3) pg/mL in the Control group and − 29.1 (− 94.1 to 43.9) pg/mL in the Dapagliflozin group. The adjusted median difference in serum Klotho levels between the variations was 52.1 (− 3.8 to 107.3) pg/mL. The ranked difference between groups, adjusted by baseline values, showed F = 4.946 (*p* = 0.03). Adjustments in the RANCOVA model for Klotho reveal that solely serum “baseline Klotho” levels were related to the variation in serum Klotho levels associated with Dapagliflozin use.

The variation in serum FGF23 levels between groups was 9.37 (− 476 to 164) pg/mL in the Control group and 20.2 (− 517 to 1143) pg/mL in the Dapagliflozin group. The adjusted median difference in serum FGF23 levels between the variations was − 179 (− 1141 to 177) pg/mL. The ranked difference between groups, adjusted by baseline values, showed F = 0.813 (*p* = 0.37).

A sensitivity analysis conducted with trimmed data (i.e., excluding extreme outliers) and with winsorized data (replacing excluded outliers with 95th percentile values) corroborated the aforementioned results (see supplementary material).

### Exploratory Analysis

#### Effects of Dapagliflozin Treatment on a Panel of Serum Bone-Related Biomarkers

The intragroup comparison of means of serum bone-related biomarkers levels at the end of 24 weeks revealed no differences (Table 2).

**Table 2:** Bone-related biomarkers profile at baseline and after 24 weeks according to the control and the dapagliflozin groups

Biochemical parameters	Control			Dapagliflozin		
	Baseline	6 months	*p*	Baseline	6 months	*p*
Total calcium (mg/dL)	9.2 (8.9–9.7)	9.1 (8.6–9.6)	0.08	9.6 (9.1–9.8)	9.4 (9.1–9.8)	0.75
Phosphate (mg/dL)	4.2 (3.5–5.0)	4.2 (3.6–5.0)	0.42	4.3 (3.3–5.3)	4.3 (3.8–5.0)	0.70
PTH (pg/mL)	108 (31–191)	110 (41–189)	1.00	65 (41–159)	92 (40–181)	0.052
Bone ALP (IU/L)	31 (22–58)	30 (20–68)	0.22	36 (19–65)	27 (21–51)	0.93
TRAP5b (IU/L)	8.2 (6–13)	8.0 (5.7–14.1)	0.57	8.7 (5.8–11.6)	9.8 (5.2–11.6)	0.18
DKK1 (pg/mL)	504 (454–925)	599 (472–905)	0.08	654 (452–1008)	795 (507–1019)	0.16
Sclerostin (pg/mL)	1514 (1097–2169)	1497 (1106–2385)	0.42	1780 (1259–2517)	1839 (1474–2539)	0.31
Osteopontin (pg/mL)	52,214 (40,482–64,842)	54,525 (39,399–77,110)	0.33	52,214 (32,421–80,524)	48,060 (28,316–67,713)	0.91
Osteoprotegerin (pg/mL)	908 (537–1270)	842 (621–1190)	0.40	824 (412–1243)	851 (446–1250)	0.28
Osteocalcin (pg/mL)	83,755 (45,604 –146,887)	93,139 (63,067–163,576)	0.61	81,788 (40,894 –143,640)	72,089 (3448 –140,662)	0.78

PTH, parathyroid hormone; ALP, alkaline phosphatase; TRAP5b, Tartrate-resistant acid phosphatase type−5b; DKK1, Dickkopf-1. Data were expressed as median and 25th and 75th percentiles. Changes from baseline within groups were compared using the paired-samples t-test or the Wilcoxon signed-rank test

The analysis between groups of the variation in all serum bone-related biomarkers between the end of the follow-up and baseline (delta) showed no significant differences. The delta for serum total calcium levels was − 0.3 (− 0.5 to 0.15) vs. 0.1 (− 0.4 to 0.3) mg/dL, *p* = 0.26; phosphate − 0.1 (− 1 to 0.6) vs. 1 (− 0.9 to 0.8) mg/dL, *p* = 0.32; PTH 12 (− 60 to 67) vs. 14 (− 13 to 82) pg/mL, *p* = 0.20; bone ALP − 4 (− 11 to 5) vs. 1 (− 10 to 5) IU/L, *p* = 0.36; TRAP5b − 0.2 (− 2.3 to 1.8) vs. 0.6 (− 1.2 to 2.4) IU/L, *p* = 0.19; DKK1 110 (− 36 to 179) vs. 46 (− 98 to 265) pg/mL, *p* = 0.84; sclerostin 47 (− 147 to 312) vs. 78 (− 242 to 370) pg/mL, *p* = 0.96; osteopontin − 3505 (− 22,694 to 10,894) vs. 4466 (− 16,574 to 14,615) pg/mL, *p* = 0.52; osteoprotegerin 44 (− 87 to 199) vs. 24 (− 92 to 188) pg/mL, *p* = 0.81; and osteocalcin 0 (− 48,431 to 42,259) vs. − 7446 (− 28,099 to 26,180) pg/mL, *p* = 0.53.

#### Effects of Dapagliflozin Treatment on Bone-Related Clinical Outcomes

Table 3 details dual-energy X-ray absorptiometry (DEXA) parameters at baseline and after 24 weeks, according to the Control and the Dapagliflozin groups. An intragroup analysis of means of bone mineral density or t-scores (including lumbar vertebrae, femoral neck, and femoral total) at 24 weeks indicated that there were no significant differences. The analysis between groups of the variation of bone mineral density or T-score (including lumbar vertebrae, femoral neck, and femoral total) between the end of the follow-up and baseline (delta), also demonstrated that there were no significant differences.

**Table 3:** Representative dual-energy X-ray absorptiometry (DEXA) parameters at baseline and after 24 weeks, according to the control and the dapagliflozin groups

DEXA	Control			Dapagliflozin		
Parameters	Baseline	6 months	*p*	Baseline	6 months	*p*
*BMD (g/cm*^*2*^*)*						
Lumbar vertebrae*	1.15 ± 0.2	1.17 ± 0.2	0.92	1.17 ± 0.2	1.21 ± 0.2	0.49
Femoral neck	0.87 ± 0.15	0.88 ± 0.16	0.90	0.87 ± 0.16	0.87 ± 0.15	0.62
Femoral total	0.90 ± 0.18	0.91 ± 0.18	0.48	0.91 ± 0.17	0.92 ± 0.16	0.45
*T-score*						
Lumbar vertebrae*	− 0.34 ± 1.7	− 0.28 ± 1.9	0.34	− 0.23 ± 1.6	0.06 ± 1.6	0.49
Femoral neck	− 1.30 ± 1.16	− 1.29 ± 1.20	0.44	− 1.32 ± 1.26	− 1.35 ± 1.21	0.35
Femoral total	− 1.08 ± 1.38	− 1.08 ± 1.41	0.18	− 1.13 ± 1.39	− 1.08 ± 1.26	0.42
*Osteopenia (N, %)*	30 (77)	24 (77)	1.00	25 (69)	20 (62)	1.00
*Osteoporosis (N, %)*	17 (44)	16 (52)	0.62	15 (42)	14 (44)	0.50

BMD, bone mineral density; *, mean values calculated from L1, L2, L3, and L4 lumbar vertebrae BMD; Data were expressed as mean ± standard deviation, or frequencies (percentages). Percentages or mean changes from baseline were compared within groups using the paired-sample t-test or McNemar‘s test; and between groups using the Wilcoxon signed-rank test or chi-square test. At baseline, N = 75; at follow-up, N = 63

The prevalence of osteopenia and osteoporosis at follow-up was 70% and 48%, respectively; 24 (77%) patients presented osteopenia in the Control group vs. 20 (62%) patients in the Dapagliflozin group (*p* = 0.27). Considering osteoporosis, 16 (52%) patients presented this diagnosis in the Control group vs. 14 (44%) patients in the Dapagliflozin group (*p* = 0.61).

After 24 weeks, no bone fractures were observed.

## Discussion

The present study explored the effects of dapagliflozin use over 24 weeks in dialysis patients on a broad panel of bone biomarkers and their related clinical outcomes. In this small and short-term study, dapagliflozin treatment seemed safe without signal of negative impacts on relevant bone-related clinical outcomes and biomarkers, besides serum Klotho levels.

A previous study aimed to evaluate the effects of other gliflozins on mineral and bone metabolism in healthy individuals [35]. The authors observed an increase in serum concentrations of phosphate, FGF23, and PTH, followed by a reduction in 1,25-dihydroxyvitamin D associated with canagliflozin. Alba et al. [36] also studied canagliflozin in patients with diabetes *mellitus*. They observed an increase in serum phosphate concentrations (5.2% in the canagliflozin group vs. 1.4% in the placebo group), without changes in PTH, 1,25-dihydroxyvitamin D, or 25-dihydroxyvitamin D, nor in mineral levels in urine. In another study [37], bone turnover markers such as carboxy-telopeptide of collagen type 1 and osteocalcin increased over 26 weeks of treatment with canagliflozin.

The reported increase in serum phosphate and FGF23 levels has been attributed to altered proximal tubular sodium handling, leading to enhanced activity of sodium–phosphate cotransporters and increased phosphate reabsorption. In contrast to these previous studies with different populations, the present study included solely patients on dialysis (with around half of them anuric), in which the absence of functional proximal tubules likely abolishes this mechanism. The lack of change in serum phosphate and FGF23 in dialysis patients, therefore, supports the notion that SGLT2 inhibitor–induced mineral alterations are kidney-dependent phenomena.

The mechanisms by which treatment with dapagliflozin results in a reduction in serum Klotho levels, as well as its clinical significance, are unknown. At first glance, the reduction in serum soluble Klotho levels seems to be a negative gliflozin-associated effect. However, it remains rather unclear how serum Klotho levels directly relate to the bone tissue levels, since its concentration in different tissues may have distinct meanings.

In an animal study [38] using a model of CKD induced by unilateral ureteral obstruction, the treatment with empagliflozin at different time points was associated with the restoration of Klotho mRNA levels in kidney tissue, reduced CKD progression, and attenuation of markers of inflammation and fibrosis.

On the other hand, the local effect of Klotho on bone appears to be more complex. Komaba et al. [39] observed that osteocyte-specific Klotho ablation caused a marked increase in bone formation and mass, and enhanced osteoblast activity. These observations are in opposition to those from studies reporting alterations in systemic Klotho, such as the studies with Klotho hippomorphic (kl/kl) mice model, which resulted in osteopenia and low bone turnover [40, 41].

While the dapagliflozin group demonstrated a statistically significant reduction in circulating Klotho, this effect was modest. Importantly, this isolated finding occurred without corresponding alterations in FGF23, phosphate, bone turnover markers, or bone mineral density. Given our relatively small sample size and the absence of concurrent physiological shifts, the clinical and biological significance of this reduction remains uncertain. Therefore, these findings must be interpreted with caution and viewed strictly as exploratory, serving primarily to generate hypotheses for larger, future long-term investigations.

In advanced CKD, skeletal fragility is driven by bone quality rather than bone quantity alone [42]. While our study found no changes in standard bone mineral density, densitometry cannot capture these qualitative dimensions. The observed reduction in serum Klotho levels following dapagliflozin treatment could theoretically impact intrinsic bone quality and osteocyte signaling without altering its mass in the analyzed time frame. Future long-term trials utilizing high-resolution peripheral quantitative computed tomography or bone biopsies are essential to fully clarify gliflozin-mediated effects on bone quality.

The effect of gliflozins on bone quantity and quality is an issue addressed by different experimental and clinical studies. Preclinical studies indicate that bone gliflozins’ effects can be influenced by sex, age, and diabetes. Long-term canagliflozin treatment in aged UM-HET3 mice compromised bone morphology and mineral composition [43]. In nondiabetic C57BL/6J mice, an intermediate period of canagliflozin treatment improved bone morphology but reduced osteoblast and bone formation markers in a sex-dependent manner [44]. In contrast, Song et al. [45] observed that canagliflozin, but not dapagliflozin nor empagliflozin, increased bone mineral density and promoted osteoblast differentiation in type 2 diabetic mice.

In clinical studies, the overall bone safety profile of the evaluated SGLT2i in patients with early CKD and diabetes is reassuring [20]. Initial safety concerns regarding fractures [15–18] have not been supported by consistently broader evidence [2–12]. However, none of these studies enrolled a significant number of patients with CKD on dialysis, a population at the highest risk for bone fractures. The data presented here indicated no impact of dapagliflozin over 24 weeks on several bone-related clinical outcomes, including fractures, osteopenia, and osteoporosis prevalence.

This study has limitations. Primarily, the 24-week follow-up period and the limited number of dialysis patients included may be insufficient to fully capture the long-term changes in bone metabolism and related clinical outcomes. In dialysis patients, meaningful BMD alterations generally require longer follow-up periods to become clinically evident. Given this temporal constraint, future long-term studies could evaluate bone tissue using advanced imaging techniques (i.e., trabecular bone score [TBS] and/or high-resolution peripheral quantitative computed tomography [HR-pQCT]) to comprehensively evaluate microarchitectural bone characteristics. Additionally, the demographic characteristics of the sample analyzed may have affected the reported results, which limits the direct application of these results to other populations. Besides, uremic toxins in dialysis patients may cause non-specific interference, affecting assay accuracy [46, 47]. This can be represented by the limitation of immunoassays to differentiate between secreted and membrane-cleaved Klotho fragments, a known issue causing inter-kit variability. Another limitation of this study is the absence of data regarding serum 25-hydroxyvitamin D [25(OH)D] levels. Vitamin D is a clinically critical parameter in CKD and shares an established, complex physiological correlation with the Klotho-FGF23 axis. Because these measurements were not collected in the original trial, we could not adjust our analyses for baseline Vitamin D status, nor evaluate its potential confounding influence on the observed reduction in serum Klotho levels associated with dapagliflozin use. Furthermore, the statistical power was limited by patient drop-off. During the 24-week follow-up, 12 patients (eight in the control group and four in the dapagliflozin group) were excluded due to loss of follow-up, transplantation, or death. Finally, this study was not powered to detect changes in BMD or fracture risk.

## Conclusions

In conclusion, dapagliflozin administered over 24 weeks in dialysis patients was independently associated with a reduction in serum Klotho levels, which was influenced solely by baseline serum Klotho levels. Dapagliflozin was not associated with other changes in biomarkers of bone metabolism or bone-related outcomes, including fracture, osteopenia, and osteoporosis. Future mechanistic and long-term clinical studies are needed to confirm bone-related outcomes and explore Klotho’s modulation by gliflozins in this population.

## Supplementary Information

Below is the link to the electronic supplementary material.

Supplementary Material 1

## Data Availability

Any additional information may be available from the corresponding author upon request.
