# Supplementary material for: Effects of Dapagliflozin on the Bone of Patients with CKD on Dialysis
Source: Calcif Tissue Int. 2026 Aug 14;117(1):130. doi: 10.1007/s00223-026-01587-7 (PMC13473233; doi:10.1007/s00223-026-01587-7)
Supplement: Supplementary file 1 [file 223_2026_1587_MOESM1_ESM.docx]

SUPPLEMENTAL FILE

Sensitivity analysis: performed before (trimmed data) and after outlier treatment (winsorized data) to confirm the observed results related to the delta variation of serum Klotho and FGF23 levels between the end of follow-up and baseline compared using ranked analysis of covariance (RANCOVA) to assess the potential effect of dapagliflozin, influenced by other factors or covariates, and adjusted by its respective baseline value.

*Delta variation of serum Klotho levels by ranked analysis of covariance (RANCOVA) with trimmed data (extreme outliers excluded)*

- Number of patients at baseline:	79
- Number of valid serum Klotho measurements at baseline:	78
- Number of extreme outliers excluded at baseline:	1
- Number of patients at follow-up:	67
- Number of valid serum Klotho measurements at follow-up:	67
- Number of extreme outliers excluded at follow-up:	2
- Number of cases (pairs) analyzed:	65

Results of RANCOVA analysis with trimmed data:

Rancova model significance: 0.001; F: 4.042; p = 0.04.

*Delta variation of serum Klotho levels by ranked analysis of covariance (RANCOVA) with winsorized data (95^th^ percentile; 5%)*

- Number of patients at baseline:	79
- Number of valid serum Klotho measurements at baseline:	78
- Number of cases replaced at baseline:	1
- Number of patients at follow-up:	67
- Number of valid serum Klotho measurements at follow-up:	67
- Number of cases replaced at follow-up:	2
- Number of cases (pairs) analyzed:	66

Results of RANCOVA analysis with winsorized data:

Rancova model significance: 0.002; F: 4.946; p = 0.03.

*Delta variation of serum FGF23 levels by ranked analysis of covariance (RANCOVA) with trimmed data (extreme outliers excluded)*

- Number of patients at baseline:	79
- Number of valid serum FGF23 measurements at baseline:	79
- Number of extreme outliers excluded at baseline:	4
- Number of patients at follow-up:	67
- Number of valid serum FGF23 measurements at follow-up:	65
- Number of extreme outliers excluded at follow-up:	5
- Number of cases (pairs) analyzed:	57

Results of RANCOVA analysis with trimmed data:

Rancova model significance: 0.01; F: 0.231; p = 0.63.

*Delta variation of serum FGF23 levels by ranked analysis of covariance (RANCOVA) with winsorized data (95^th^ percentile; 5%)*

- Number of patients at baseline:	79
- Number of valid serum FGF23 measurements at baseline:	79
- Number of cases replaced at baseline:	4
- Number of patients at follow-up:	67
- Number of valid serum FGF23 measurements at follow-up:	65
- Number of cases replaced at follow-up:	5
- Number of cases (pairs) analyzed:	65

Results of RANCOVA analysis with winsorized data:

Rancova model significance: 0.03; F: 0.813; p = 0.37.
